# COVID-19 Vaccination is not a Sufficient Public Policy to face Crisis Management of next Pandemic Threats

**DOI:** 10.1007/s11115-022-00661-6

**Published:** 2022-10-17

**Authors:** Mario Coccia

**Affiliations:** CNR -- NATIONAL RESEARCH COUNCIL OF ITALY, Collegio Carlo Alberto, Via Real Collegio, n. 30, 10024 Moncalieri (TO), Italy

**Keywords:** COVID-19 pandemic, COVID-19 mortality, Vaccination, Public policies, Health policy, Variants, Peltzman effect

## Abstract

This study reveals that a vast vaccination campaign is a necessary but not sufficient public policy to reduce the negative impact of Coronavirus Disease 2019 (COVID-19) pandemic crisis because manifold factors guide the spread of this new infectious disease and related mortality in society. Statistical evidence here, based on a worldwide sample of countries, shows a positive correlation between people fully vaccinated and COVID-19 mortality (*r* = + 0.65, p-value < 0.01). Multivariate regression, controlling income per capita, confirms this finding. Results suggest that the increasing share of people vaccinated against COVID-19 seems to be a necessary but not sufficient health policy to reduce mortality of COVID-19. The findings here can be explained with the role of Peltzman effect, new variants, environmental and socioeconomic factors that affect the diffusion and negative impact of COVID-19 pandemic in society. This study extends the knowledge in this research field to design effective public policies of crisis management for facing next pandemic threats.

## Introduction

In 2022 we are still in the throes of negative socioeconomic effects of the pandemic of Coronavirus Disease 2019 (COVID-19), an infectious illness generated by new viral agent of the Severe Acute Respiratory Syndrome Coronavirus 2 or SARS-CoV-2 (Chowdhury et al., 2022; Coccia, 2020, 2021; Coccia, 2021a; Coccia, 2022; Bontempi et al., 2021; Johns Hopkins Center for System Science and Engineering, 2022; Núñez-Delgado et al., 2021; Vinceti et al., 2021). Initially, in 2020, countries implement non-pharmaceutical measures of control (e.g., lockdown, quarantine, etc.) to cope with COVID-19 pandemic crisis (Askitas et al., 2021; Biswas & Alfandari, 2022; Coccia, 2021, 2021b, 2021d; Benati & Coccia, 2022). Mahmoudi & Xiong (2022) argue that lower rates of COVID-19 infection and mortality are associated with appropriate mitigation and containment policies. In 2021 and 2022, the most applied health policy worldwide to face COVID-19 is the administration of new types of vaccines based on viral vector, protein subunit and nucleic acid-RNA (Abbasi, 2020; Coccia, 2022a, 2022b, 2022c; Mayo Clinic, 2021).

Vaccination plans endeavor to reduce the diffusion of COVID-19 in order to relax non-pharmaceutical measures of control and maintain a low reproduction number (Aldila et al., 2021; Coccia 2022c, 2022d; Moore et al., 2021). Akamatsu et al. (2021) argue that an efficient campaign of vaccination can substantially reduce infections in society and avoid the collapse of health sector (cf., Benati & Coccia 2022a; Coccia, 2022a). Shattock et al. (2022) point out that a rapid rollout of vaccination can allow the relaxation of non-pharmaceutical interventions, but emerging variants of SARS-CoV-2 create new scenarios and problems for epidemic control (Fontanet et al., 2021; Khandia et al., 2022; Papanikolaou et al., 2022).  Shattock et al. (2022) also show that a timely vaccination can reduce the size of pandemic wave. Benati & Coccia (2022a) suggest the positive effects of good governance for supporting a timely COVID-19 vaccination directed to reduce pandemic impact in countries. Aldila et al. (2021) maintain that higher levels of vaccination can mitigate the diffusion of COVID-19 and increase the probability of achieving the herd immunity to protect vulnerable individuals (cf., Anderson et al., 2020; de Vlas & Coffeng, 2021; Randolph & Barreiro, 2020). However, Aschwanden (2020, 2021) raises many doubts about the goal of reaching the herd immunity for COVID-19 with new vaccines: herd immunity in the presence of COVID-19 is a “false promise” because new variants and other factors affect transmission dynamics and mortality of COVID-19  in society (cf., Ackley et al., 2022; Davies et al., 2021).

In this context, an important problem is the evaluation of the effectiveness of public policies based on vaccination for reducing high numbers of COVID-19 related deaths between countries, and as a consequence, to control the pandemic impact in society (Saadi et al., 2021). In particular, the understanding of the effectiveness of COVID-19 vaccination and the identification of driving factors concerning the diffusion and mortality of COVID-19 are critical aspects for planning effective policy responses to cope with next pandemic crises (Garber, 2021; Islam et al., 2021; Sanmarchi et al., 2021; Stokes et al., 2021, 2021a).

  Hence, in the presence of necessity to design appropriate health policy to cope with COVID-19 pandemic crisis, the goal of this paper is to clarify the following problem:

*are policy responses based on vast vaccination a sufficient strategy to reduce mortality of COVID-19 in society?*

This study endeavors to expand the understanding of this problem to design effective public policies of crisis management to cope with next pandemic threats (Farazmand, 2001, 2014).

## Study Design and Methodology

### Sample

The total sample of this study is *N* = 151 countries worldwide. The sample can have a lower size in some statistical analyses for missing data of variables (cf., Coccia, 2018).

### Measures for Statistical Analyses

- Vaccination is measured with the percent share of people fully vaccinated against COVID-19 at the 11 January 2022. Data of countries refer mainly to January 2022 but some countries, because of the difficulty in the gather and transmission of information, can have data related to December 2021. Of course, this aspect does not affect the statistical analyses that are based on a large sample with more than 100 countries. Data here consider all types of COVID-19 vaccines used in countries, i.e.: vaccines by Johnson & Johnson, Oxford/AstraZeneca, Pfizer/BioNTech, Sinopharm/Beijing, Sinovac, Sputnik V and Moderna (Ritchie et al., 2020). Of course, every country uses a different combination of these COVID-19 vaccines to protect the population. Dataset is based on the most recent official numbers from governments and health ministries worldwide. Source: Our World in Data, 2022, 2022a, Ritchie et al., 2020.
- Gross Domestic Product (GDP) per capita in 2020 (constant US$2010). GDP is the sum of gross value added by all resident producers in the economy plus any product taxes and minus any subsidies not included in the value of products. Source: The World Bank (2022).
- Population in 2020. Total population includes all residents regardless of legal status or citizenship. Source: The World Bank (2022a).
- COVID-19 Deaths. Total number of deaths attributed to COVID-19 in January 2022. It indicates the severity of this novel infectious disease (i.e., COVID-19) in socioeconomic systems (Angelopoulos et al., 2020). This study calculates the mortality ratio of COVID-19 per 100,000 people to have a normalized value for a comparative analysis between countries (Coccia, 2018; Benati & Coccia, 2019) given by:

$$\begin{array}{l}Mortality\ {\rm{ }}ratio\ {\rm{ }}of\ {\rm{ }}COVID - 19\ {\rm{ }}per\ {\rm{ }}100,000\ {\rm{ }}people = \\\left( {\frac{{Total\ {\rm{ }}Number\ {\rm{ }}of\ {\rm{ }}Deaths\ {\rm{ }}from\ {\rm{ }}COVID - 19\ {\rm{ }}at\ {\rm{ }}January\ {\rm{ }}2022}}{{Total\ {\rm{ }}Population\ {\rm{ }}in\ {\rm{ }}2020}}} \right) \times 100,000\ {\rm{ }}Inhabitants\end{array}$$

Source: Johns Hopkins Center for System Science and Engineering (2022).

### Model and data Analysis Procedure

Firstly, data are analyzed with descriptive statistics (arithmetic mean and standard error of the mean). The normal distribution of variables under study is checked with skewness and kurtosis coefficients. If variables have not a normal distribution, variables are transformed in logarithmic scale for having normality and performing appropriate parametric analyses.

Secondly, bivariate Pearson Correlation measures the strength and direction of linear relationship between share % of people fully vaccinated against COVID-19 and mortality ratio of COVID-19 per 100,000 people. This study also measures the strength and direction of the linear relationship between mortality ratio of COVID-19 and share % of people fully vaccinated by controlling the effect of GDP per capita (analysis of partial correlation).

Thirdly, multiple regression analyzes the mortality ratio of COVID-19 (*dependent* or *response variable*) on two explanatory variables: share % of people fully vaccinated against COVID-19 and GDP per capita (*predictors*).

The specification of *log*-*log* model is:

log$${y}_{i,t}={\alpha }_{0}+{\beta }_{1}\text{ }\text{log }{\text{x}}_{\text{i,t}}+ {\beta }_{2}\text{ log z}{{\text{ }}_{\text{i,t-}\text{2}}^{}}_{}+{u}_{i,t}\hspace{1em}$$ [1]

where:

- *y*_*i, t*_ = Mortality ratio attributed to COVID-19 in January 2022.
- *x*_*i,t*_ = Share % of people fully vaccinated against COVID-19 in January 2022.
- *z*_*i, t−2*_ = GDP per capita in 2020.
- *u*_*i,t*_ = Error term.

Country *i = 1, …, n; t = time*.

Results of regression analysis are the R^2^ and standard error of the estimate. R^2^ is the coefficient of determination and indicates the proportion of variance in the dependent variable that can be explained by independent variables. The *F* test assesses if the overall regression model has a good fit for data. Unstandardized coefficients of partial regression indicate how much the dependent variable varies with an independent variable, when the other independent variable is held constant; finally, the statistical significance of parameters is based on *t*-test.

Statistical analyses are performed with the Statistics Software SPSS-version 26.

## Results

**Table 1:** Descriptive statistics of sample

Variables	N	Mean	Std. Error of Mean	Skewness	Kurtosis
GDPPC2020, GDP per capita	151	$14,457.69	$1,716.74	2.68	9.64
MOR2022, Mortality ratio of COVID-19 per 100,000 people	151	111.43	9.75	1.33	1.69
VAC2022, Share % of people fully vaccinated against COVID-19	144	44.14	2.26	-0.13	-1.29
Log GDPPC2020	149	8.68	0.12	0.07	-0.90
Log MOR2022	151	3.82	0.13	-0.58	-0.68
Log VAC2022	144	3.40	0.09	-1.44	1.39

*Note*: sample size N changes for missing values in some countries

Table 1 shows descriptive statistics of the sample under study and that variables with logarithmic transformation have a normal distribution (coefficients of skewness and kurtosis have values in the correct range) to perform appropriate and robust parametric analyses.

**Table 2:** Correlation

♣ Bivariate correlation		LogVAC2022	LogMOR2022
N = 144	LogVAC2022	1	0.646**
♣ Partial Correlation			
*Control variable*: GDPPC2020		LogVAC2022	LogMOR2022
N = 135	LogVAC2022	1	0.443***

*Note*: MOR2022 = COVID-19 Mortality ratio per 100,000 people in 2022; VAC2022 = share % of people fully vaccinated against COVID-19 in 2022. GDPPC 2020 = GDP per capita in 2020

Sample size N changes for missing values in some countries.

*** Correlation is significant at the 0.001 level (1-tailed).

** Correlation is significant at the 0.01 level (1-tailed).

The bivariate correlation reveals a positive coefficient *r*_bivariate correlation_ =0.65 (*p*-value < 0.01), which indicates a strong correlation between COVID-19 mortality ratio per 100,000 people and share % of people fully vaccinated against COVID-19 (Table 2). This finding is confirmed in Table 2 with the partial correlation that indicates the moderate linear relationship between variables just mentioned, controlling the effect of GDP per capita (*r*_partial correlation_ = 0.44, *p*-value < 0.001).

**Table 3:** Regression analyses of COVID-19 mortality ratio in 2022 on people fully vaccinated in 2022 (and on GDP per capita 2020), *log-log* model [1]

	Simple Regression	Multiple regression
Constant α (St. Err)	0.754* (0.325)	−0.542 (0.665)
VAC2022, Coefficient β_1_ (St. Err.)	0.917*** (0.091)	0.713*** (0.132)
GDPPC2020, Coefficient β_2_ (St. Err.)	--	0.228* (0.103)
R^2^ (St. Err. of Estimate)	0.42 (1.23)	0.43 (1.22)
*F-test*	101.70***	52.80***

*Note*: Dependent (response) variable is: MOR2022 = COVID-19 Mortality ratio per 100,000 people in 2022; Explanatory variables are: VAC2022 = share % of people fully vaccinated against COVID-19 in 2022 and GDPPC2020 = Gross Domestic Product per capita in 2020. Significance: ***=p-value < 0.001; *=*p-*value < 0.05

**Fig. 1:**
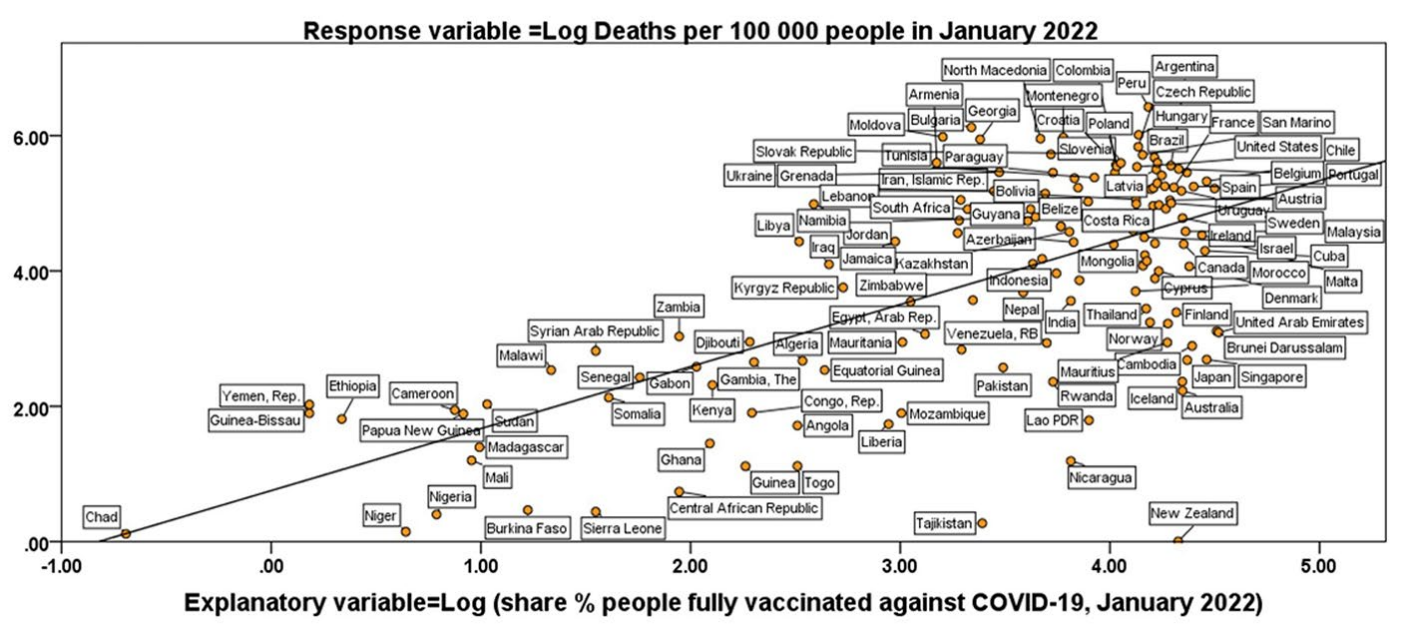
Relation of COVID-19 mortality ratio per 100,000 people on share % of people vaccinated against COVID-19 based on *log*-*log* model

Table 3 shows the results of simple and multiple regression. Since these statistical analyses provide rather similar results, we describe the estimated multivariate relationship based on Eq. [1] with two explanatory variables (i.e., Share % of people fully vaccinated against COVID-19 in 2022 and Gross Domestic Product per capita in 2020). The partial coefficient of regression β_1_ of the model indicates that a 1% higher share of people fully vaccinated (controlling GDP per capita), increases the expected mortality ratio of COVID-19 per 100,000 people by 0.7% (*p*-value < 0.001), whereas the partial coefficient of regression β_2_ of the model indicates that a 1% higher level of GDP per capita (controlling share % of people fully vaccinated), increases the expected mortality ratio of COVID-19 per 100,000 people by about 0.3% (*p*-value < 0.05). *F*- test (the ratio of the variance explained by the model to the unexplained variance) is significant at 1‰, such that overall regression analysis provides a good fit of data. R^2^ of the model of multiple regression indicates that about 43% of the variation in mortality ratio of COVID-19 can be attributed (linearly) to share % of people fully vaccinated against COVID-19 in 2022 and Gross Domestic Product per capita in 2020. Figure 1 shows regression line of COVID-19 mortality ratio per 100,000 people on share % of people vaccinated against COVID-19, based on *log*-*log* model.

Kim & Lee (2022) argue that COVID-19 related infections and deaths are both negatively associated with the fraction of the population vaccinated with at least one dose. These scholars support, in the short term, the heath policy of extending the interval between first and second dose of administering COVID-19 vaccines associated with non-pharmaceutical measures of control. Qiu et al. (2022) maintain that restriction policies are effective responses to reduce COVID-19 transmission and should remain, temporarily, also with vaccination campaigns. However, Zhu & Tan (2022) analyze the effectiveness of Hong Kong’s strict border restrictions with mainland China in curbing the transmission of COVID-19. Results suggest that border restriction policy and its further extension may not be a useful health policy in containing the spread of COVID-19, when the novel coronavirus is circulating in society; furthermore, restriction polices increase economic and social costs.

The result of the study here opens alternative explanations of the effectiveness of COVID-19 vaccination on world-wide scale and long run as will be discussed in next section.

## Phenomena Explained

**Fig. 2:**
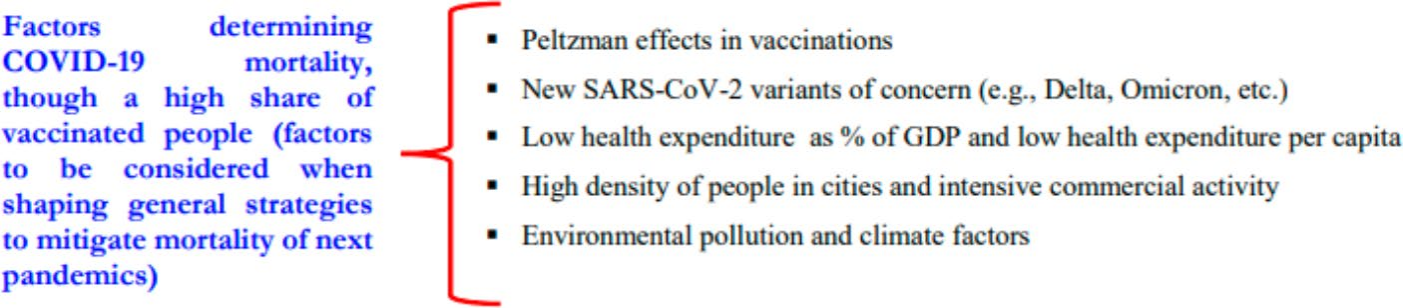
Factors affecting mortality ratio of COVID-19, though a high share of vaccinated people between countries. Factors to be considered to design general guidelines to constrain pandemic crises of novel viral agents like Severe Acute Respiratory Syndrome Coronavirus 2 (SARS-CoV-2)

Results here suggest that the increasing share of people vaccinated against COVID-19 seems to be a necessary but not sufficient health policy to reduce mortality of COVID-19 pandemic in society. This finding can be due to the mutant viral agent of SARS-CoV-2 that generates high transmissibility and other environmental and socioeconomic factors that support the diffusion of COVID-19 and reduce the effectiveness of new vaccines (Coccia, 2020 ; Coccia, 2020a, 2021a). Figure 2 systematizes some factors that may increase the COVID-19 mortality ratio between countries, though a high share of vaccination in society. These aspects should be considered to design effective public policy to cope with next pandemic crisis in order to reduce the negative impact of new infectious diseases in society when countries roll out vaccination plans.

Hence, although a high share % of COVID-19 vaccination, the following factors can increase negative pandemic impact and mortality: 

### □ Peltzman Effects in Vaccinations

Empirical results of the study here can be explained with the Peltzman theory (Peltzman, 1975). In general, medical treatments with vaccines can certainly help to lower risk of serious effects of COVID-19 and high numbers of deaths in the short run, but the Peltzman theory predicts that when safety measures are implemented, such as vaccination campaign, people also tend to increase their risky behavior (e.g., not wearing facemasks, attending crowded places, etc.). This social behavior can be due to a lower vaccinated people’s perception of risk to be infected of COVID-19, and as a consequence vaccinated people make riskier decisions and behavior that increase the widespread of the viral agent in population, especially in the presence of new variants that spread more easily between people, increasing the number of infections and also deaths  (cf., Prasad & Jena 2014). Therefore, Peltzman theory predicts that (mandatory or not) safety measures based on vaccination can generate a lower benefit than expectation because the safety benefits of these measures are offset to some extent by increases in the risky behavior of vaccinated people, in particular when new vaccines have low effectiveness to cope with variants (Iyengar et al., 2022).

### □ New SARS-CoV-2 Variants of Concern

The novel coronavirus (SARS-CoV-2) in environment constantly changes through mutations that generate variants and reduce the effectiveness of vaccines (Fontanet et al., 2021).  Davies et al. ( 2021) show that the Alpha variant (B.1.1.7) of SARS-CoV-2 was more transmissible than pre-existing variants and the hazard of deaths associated with B.1.1.7 was 61% higher than previous variants. Other two variants of the novel coronavirus that cause health and socioeconomic problems, reducing the effectiveness of COVID-19 vaccines without a calibration process in drug development process for facing rapid mutations of viral agent (Mayo Clinic, 2022), are:

- Delta (B.1.617.2). This variant was nearly twice as contagious as earlier variants and caused a more severe illness. People that are fully vaccinated can spread the virus to others. Research suggests that COVID-19 vaccines are slightly less effective against the Delta variant.
- Omicron variants (BA1, BA2, BA3, BA4, BA5 and BA.2 + L452X). These variants might spread more easily than other variants, including Delta. It is expected that people who are fully vaccinated can spread the mutant coronavirus to others. These variants also reduce the effectiveness of vaccines and of some monoclonal antibody treatments (Khandia et al., 2022).

Hence, these variants of the novel coronavirus change the transmissions dynamics of COVID-19 and the effectiveness of vaccines, generating unforeseen negative effects in society.

### □ Health Investments

Coccia (2021) reveals that countries with lower fatality rates of COVID-19 have a high average level of health expenditure (7.6% of GDP) and high average government health expenditure per capita (about $2,300); instead, countries with higher fatality rates of COVID-19 have an average health expenditure of roughly 6% of GDP and a very low government health expenditure per capita (about $243 per inhabitants) that indicate a weak healthcare sector to cope with pandemics and also other diseases in society. Kapitsinis (2020) argues that investments in health sector are a critical public policy to mitigate mortality of COVID-19 and also of other diseases. Hence, countries should support economic development and institution change direct to increase resources and incentives for healthcare investments that foster new knowledge, innovative drugs and path-breaking medical technologies that can counteract future pandemic threats like COVID-19 (Ardito et al., 2021; Coccia & Finardi, 2013; Mosleh et al., 2022; Coccia & Rolfo, 2000; Pagliaro & Coccia, 2021; Coccia and Bellitto, 2018; Coccia, 2017; Coccia, 2018a, 2018b, 2019; Coccia & Benati 2018).

### □ High Density of Cities and Intensive Commercial Activities

Coccia (2020) shows, with an Italian case study, that average number of COVID-19 infected individuals increases with average density of people/km^2^. Moreover, Bontempi & Coccia (2021) find out that an intensive commercial activity, measured with the level of import and export, can explain the diffusion of COVID-19 in society. In fact, Bontempi et al. (2021) analyze three large countries in Europe (Italy, France, and Spain) and suggest the positive association between trade and pandemic diffusion. In general, international trade fosters many drivers of the COVID-19 transmission and of other infectious diseases that are related to economic, demographic and environmental aspects. Hence, high density in cities and intensive commercial trade increase the mobility of people, diffusion of the COVID-19 and its variants and affect the effectiveness of vaccination and level of mortality attributed to COVID-19.

### □ Environmental Pollution and Climate Factors

Finally, environmental pollution and climate factors play a vital role in the transmission of COVID-19 and affect the effectiveness of vaccinations and mortality related to COVID-19. Coccia (2020) reveals in Italy that higher infections of COVID-19 were in cities with more than 100 days per year exceeding limits set for PM_10_ or ozone and cities located in hinterland zones with low average wind speed and low average temperature. The combination of these factors (environmental pollution, particulate matter, etc.) affects the immune system of people (Coccia, 2020). Studies show that sustainable environments, based on renewable energy in socioeconomic systems, can reduce air pollution and factors driving COVID-19 related infected individuals and deaths (Coccia, 2020b, 2021; Chirumbolo et al., 2022; Copat et al., 2020; Pronti & Coccia, 2021).

As far as climate factors are concerned, Rosario et al. (2020, p. 4) suggest that the exposure of the novel coronavirus to solar radiation reduces the diffusion of COVID-19. Nicastro et al. (2021) also show that Ultraviolet A and B rays have a powerful virucidal effect on the single-stranded RNA virus of the COVID-19. In fact, the solar radiation that reaches temperate regions of the Earth at noon during summers, it is a sufficient condition to inactivate 63% of virions in open-space concentrations in less than 2 minutes. Hence, low air pollution, hot temperature, low humidity and high wind speed are environmental aspects that can improve sustainability and benefits for immune system of people. These aspects reduce the circulation of COVID-19 and related mortality, supporting the effectiveness of vaccination (Coccia, 2020, 2020a, 2021a).

## Conclusion

Lau et al. (2021) argue that in the presence of a continuous global pandemic threat, the mortality ratio is a main indicator to evaluate the effectiveness of containment and vaccination policies (cf., Liu et al., 2021). In this context, one of the goals of nations, to cope with COVID-19 pandemic crisis, is the reduction of mortality related to COVID-19 (Coccia, 2021, 2021c, 2021e, 2021f; Coccia, 2022e). In 2021–2022 period, the most applied health policy worldwide, to mitigate negative effects of COVID-19, is the vaccination of vast populations (Coccia, 2022a, 2022b, 2022c). Findings here reveal that the increase of vaccinated people (%) against COVID-19 is a necessary but not sufficient health policy of pandemic control. To put it differently, a vast vaccination plan, with new vaccines,  is not associated with a general reduction of mortality of COVID-19 between countries because manifold factors can affect the diffusion and negative impact of COVID-19 pandemic in society (Seligman et al., 2021).  Chirumbolo et al. (2022) argue that the scientific community should extend the knowledge in these research fields to support decision making of policymakers for improving the crisis management of next pandemics.

What this study adds is:

- Vast vaccination campaign is a necessary but not sufficient public policy to reduce the negative impact of COVID-19 mortality worldwide because of manifold factors that guide the spread and negative effects of this new infectious disease in socioeconomic systems.
- Uncertain effects of high level of vaccination in curbing the mortality of COVID-19 can be due to new variants of the viral agent, such as Delta and Omicron.
- Role of Peltzman effect and of manifold socioeconomic and environmental factors that can explain the lower benefit of COVID-19 vaccination than expectation.
- Preparedness of countries to cope with next pandemics can improve if based on high health expenditure, high investments in new technology and a good public governance.

Although this study has provided interesting results, that are of course tentative, it has also limitations. First, a limitation of the study is the lack of data about total vaccination in manifold countries. Second, not all confounding factors that affect the diffusion and mortality of COVID-19 are taken into consideration and in future studies these factors have to be analyzed for supporting results here. Finally, the extension of the period under study and the update of data are needed to improve the understanding of these topics, reinforce results of statistical analyses and to truly warrant policy conclusions for crisis management of next pandemics.

Overall, then, despite these limitations, results here seem to show that the vaccination plans based on new vaccines are a necessary but not sufficient health policy to reduce mortality of COVID-19 in society. This study suggests that the public policy of countries, to cope with next pandemics and epidemics, should be based on different factors and disciplines that are not only related to medicine but also to social, economic, sustainable, environmental and innovation sciences. Hence, the planning of a comprehensive and multidisciplinary strategy of containment can increase the effectiveness of policy responses to face next pandemic crisis (Coccia, 2022; Farazmand, 2001, 2014). To conclude, socio-economic and public organization factors, and not only medical ones, should shape and support a general public policy based on a good governance, high health investments and new medical technology to improve the preparedness of nations to face future pandemic threats.

## Data Availability

Johns Hopkins Center for System Science and Engineering, 2022. Coronavirus COVID-19 Global Cases, https://gisanddata.maps.arcgis.com/apps/opsdashboard/index.html#/bda7594740fd40299423467b48e9ecf6 (accessed in 14 January 2022). Our World in Data 2022. Coronavirus (COVID-19) Vaccinations - Statistics and Research - Our World in Data https://ourworldindata.org/covid-vaccinations (accessed 25 January 2022).
